# Two-Sample Mendelian Randomization detects bidirectional causality between gut microbiota and celiac disease in individuals with high genetic risk

**DOI:** 10.3389/fimmu.2023.1082862

**Published:** 2023-06-30

**Authors:** Bárbara P. González-García, Sergi Marí, Ariadna Cilleros-Portet, Alba Hernangomez-Laderas, Nora Fernandez-Jimenez, Iraia García-Santisteban, Jose Ramon Bilbao

**Affiliations:** ^1^ Department of Genetics, Physical Anthropology and Animal Physiology, University of the Basque Country (UPV/EHU) and Biocruces Bizkaia Health Research Institute, Leioa, Spain; ^2^ Spanish Biomedical Research Center in Diabetes and Associated Metabolic Disorders (CIBERDEM), Madrid, Spain

**Keywords:** Mendelian randomization, celiac disease, HLA-DQ2, gut microbiota, bidirectional causality

## Abstract

**Background:**

Celiac Disease (CeD) is an autoimmune disorder triggered by gluten intake in genetically susceptible individuals. Highest risk individuals are homozygous for the Human Leucocyte Antigen (HLA) DQ2.5 haplotype or DQ2.5/DQ2.2 heterozygous. Both the HLA-DQ2-positive high genetic risk individuals and those that have developed the disease have altered intestinal microbiota, but it remains unclear whether these alterations are a cause or a consequence of CeD.

**Objective:**

To investigate a potential bidirectional causality between gut microbiota (GM) and CeD in HLA-DQ2 high genetic risk individuals.

**Materials and Methods:**

We performed a bidirectional Two-Sample Mendelian Randomization (2SMR) test using summary statistics from the largest publicly available Genome-Wide Association Study (GWAS) of GM and the summary statistics of the Immunochip CeD study of those individuals with the HLA-DQ2 high-risk haplotype. To test whether changes in GM composition were causally linked to CeD, GM data were used as exposure and CeD data as outcome; to test for reverse causation, the exposure and outcome datasets were inverted.

**Results:**

We identified several bacteria from *Ruminococcaceae* and *Lachnospiraceae* families of the Firmicutes phylum as potentially causal in both directions. In addition, our results suggest that changes in the abundance of *Veillonellaceae* family might be causal in the development of CeD, while alterations in *Pasteurellaceae* family might be a consequence of the disease itself.

**Conclusion:**

Our results suggest that the relationship between GM and HLA-DQ2 high risk individuals is highly complex and bidirectional.

## 1 Introduction

Celiac Disease (CeD) is an autoimmune disorder caused by gluten intake that develops in genetically susceptible individuals (1). The genetic susceptibility to CeD arises principally from Human Leukocyte Antigen (HLA) genes (2). More than 90% of celiac patients present the HLA-DQ2 heterodimer, encoded in *cis* by the HLA-DQA1*05:01 and DQB1*02:01 alleles that form the DQ2.5 haplotype (3). Among them, those with two copies of the HLA-DQ2 heterodimer (homozygous for the HLA-DQ2.5 haplotype or heterozygous for haplotypes HLA-DQ2.5 and HLA-DQ2.2, formed by alleles HLA-DQA1*02:01 and DQB1*02:02) are at the highest genetic risk of developing the disease (4). However, not all individuals carrying the HLA-DQ risk alleles finally develop CeD, suggesting that additional factors are needed to trigger disease onset.

In recent years, several studies have suggested that an additional factor involved in the pathogenesis of CeD could be the gut microbiota (5). The gut microbiota (GM) is defined as the community of microorganisms that live in the human gastrointestinal tract. The core GM is shaped early in life and is strongly influenced by multiple environmental factors such as mode of delivery, type of milk feeding (6), diet, antibiotic consumption (7), and also, by host genetics (8). At approximately three years of age, a relative stability in GM composition and diversity is reached, with phyla Firmicutes and Bacteroidetes representing 90% of all bacteria (9); these, together with other less abundant phyla such as Proteobacteria, Actinobacteria and Verrucomicrobia shape the healthy GM (10).

Cross-sectional studies in children and adults have shown that celiac patients have an altered composition of intestinal microbiota compared to non-celiac individuals (11–13). However, it must be mentioned that a clear microbiome signature has not been evidenced yet, in part due to the heterogeneity of the experimental procedures and design of these kind of studies (14). Moreover, the control subjects enrolled in these kinds of observational studies might suffer from other pathologies that could cause microbiota alterations. Another important limitation is that the majority of celiac patients are on a gluten-free diet (GFD) at the time of the study, and gluten is a crucial factor that *per se* influences GM composition, and thus constitutes an inevitable confounder (15–17). Prospective studies that have followed up cohorts of infants at risk of developing CeD have reported that the HLA-DQ genotype itself influences intestinal microbiota composition (18–22). While these studies provide valuable information, they are often restricted to very few individuals, since not all the HLA-DQ risk subjects finally develop CeD. Finally, it is unclear whether the alterations in the microbial community observed in celiac patients are a cause or a consequence of the disease (23).

Mendelian Randomization (MR) is an alternative approach that overcomes the limitations of observational studies, such as confounding factors or reverse causation. MR uses Single Nucleotide Polymorphisms (SNPs), that are randomly and independently allocated to each individual at conception, as instrumental variables to assess a potential causal relationship between an exposure and an outcome (24). Following this approach, our group identified a number of genetic variants associated with bacterial composition and function that might be driving CeD pathogenesis (25). More recently, using larger GM genomic datasets, other groups have performed bidirectional MR analyses to infer causal effects of gut microbial abundance on CeD risk, and *vice versa* (8, 26–28). However, no previous study has evaluated the potential bidirectional causality between GM composition and CeD in the context of the high-risk HLA (HR-HLA) haplotypes.

In the present work, we aim to explore whether the GM composition contributes to the development of CeD in HLA-DQ2 high genetic risk individuals, or if the disease itself is what modifies the composition of the GM in those genetically predisposed individuals (29).

## 2 Materials and methods

### 2.1 GWAS data sources

Two GWAS datasets were used for the Mendelian Randomization analysis. On the one hand, summary statistics from the MiBioGen microbiome consortium GWAS meta-analysis (8) were downloaded from www.mibiogen.org, and directly used for our analysis. This study represents the largest microbiome GWAS up to date including 18,340 individuals from 24 cohorts from different ancestries: 72.3% European, 6.0% American Hispanic/Latin, 4.4% East Asian, 2.6% from Middle Eastern countries, 0,7% African American and 14.0% from multiple ancestries. This large study analyzes the association of the host genotype with a total of 211 microbial taxa (9 phyla, 16 classes, 20 orders, 35 families, and 131 genera). On the other hand, individual-level genotype data from the Immunochip CeD study (29) were reanalyzed selecting only HR-HLA individuals, as described in the following section. The Immunochip is a case-control study that aimed to identify non-HLA risk *loci* associated with CeD by genotyping 12,041 CeD cases and 12,228 healthy controls (97.5% of the individuals were of European ancestry, and 2.5% from India).

### 2.2 Quality control, genotype imputation and genome-wide association analysis

The genotypes from the Immunochip CeD dataset were subjected to SNP and sample quality controls. SNPs with a Minor Allele Frequency (MAF) below 1%, a call rate below 95%, or a Hardy-Weinberg equilibrium *p-*value under 1x10^-6^ were discarded. In addition, samples with a call rate below 97%, sex discrepancies and those with heterozygosity levels higher than four times the standard deviation of the mean were also excluded.

In order to select HR-HLA individuals, DQ haplotypes were imputed with the *HIBAG-HLA Genotype Imputation* R package (*Version 1.32.0*) (30) and 4,814 individuals (4,264 celiac patients and 550 controls), either homozygous for HLA-DQ2.5 (HLA-DQA1*05_01-DQB1*02:01) or heterozygous for HLA-DQ2.5/DQ2.2 (HLA-DQA1*05:01-DQB1*02:01/DQA1*02:01-DQB1*02:02) were selected. Genome-wide imputation of HR-HLA individuals was carried out in the Michigan Imputation Server (31) using the TOPMed Imputation Reference panel (32) with the GRCh38 genomic coordinates. Variants with an R^2^ imputation accuracy value lower than 0.9 were filtered out.

The association analysis was performed using SNPTEST (*Version 2.5.6*) (33), with score test and an additive model. A Manhattan plot was built using the *qqman* R package (*Version 0.1.8*) (34).

### 2.3 Bidirectional two-sample mendelian randomization

**Figure 1:**
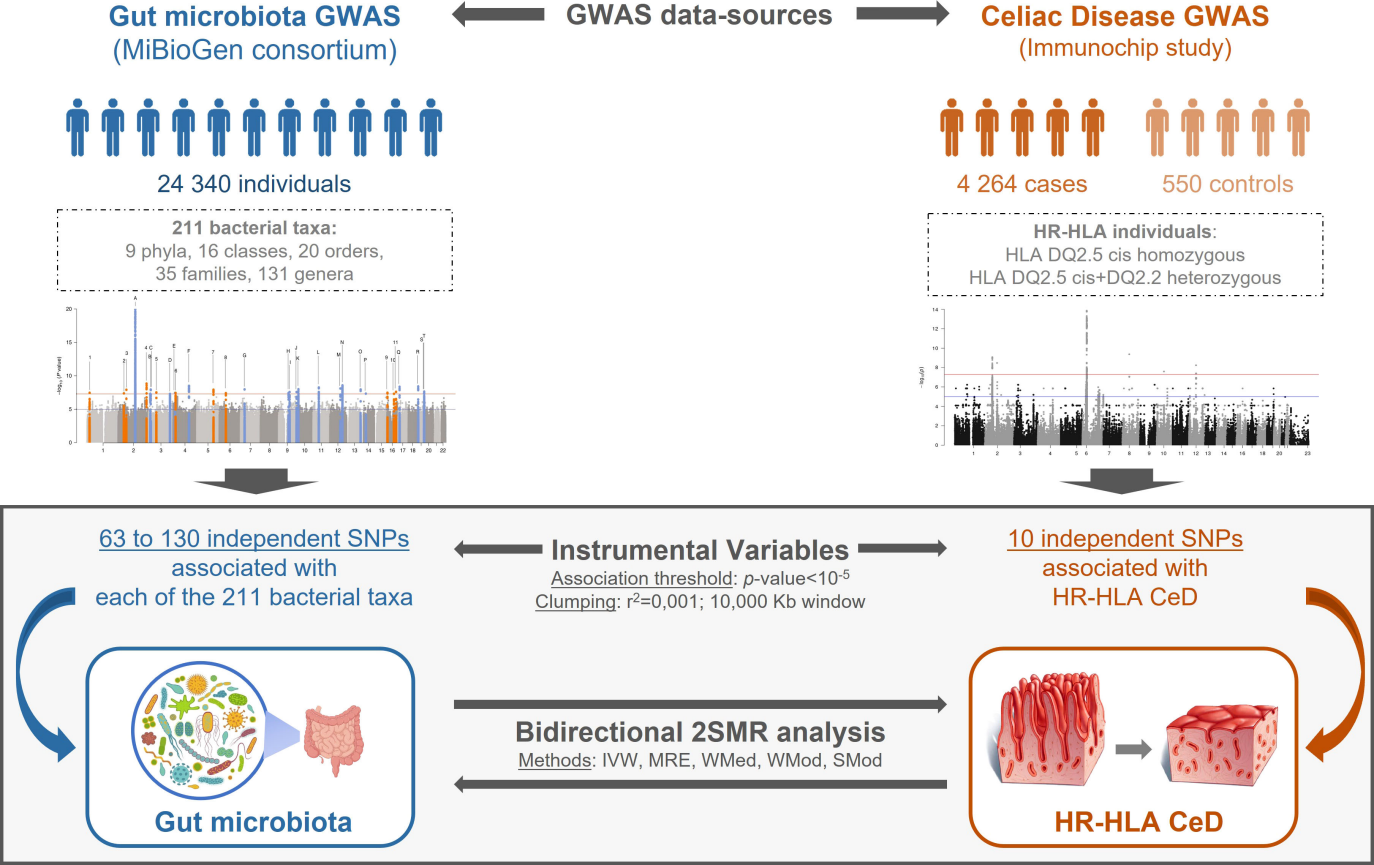
Schematic representation of the bidirectional 2SMR study. GM and CeD datasets were obtained from MiBioGen consortium and Immunochip study, respectively. GM dataset included genotype association of 211 taxa in 18,340 individuals; the CeD Immunochip was reanalyzed to only include genetic associations of HR-HLA individuals. Instrumental variables from exposure data were selected applying a *p*-value<10^-5^ association threshold, a pairwise LD r^2^<0.001 and a 10,000 kb clumping window. The bidirectional 2SMR analysis: GM > HR-HLA CeD or HR-HLA CeD > GM was performed using five methods: Inverse Variance Weighted (IVW), MR Egger (MRE), Weighted Median (WMed), Weighted Mode (WMod), and Simple Mode (SMod).

The reciprocal causality between GM and HR-HLA CeD was assessed following a bidirectional 2SMR approach (**Figure 1**) using the *TwoSampleMR* R package (*Version 4.26*) (35). To test if changes in GM composition were causally linked to CeD in HR-HLA individuals, public data from MiBioGen Consortium (n=18,340) were used as exposure data, and the association study results (n=4,814) from the HR-HLA Immunochip study were used as outcome data. To test for reverse causation, the exposure and outcome datasets were inverted.

Exposure data were prepared using the *format_data()* function. For Instrumental variables (IVs) associated with bacterial taxonomies (phylum, class, order, family, or genus) a nominal threshold *p*-value of 1x10^-5^ was chosen (as this cut-off value was previously used in analogous 2SMR studies by the MiBioGen consortium to extract sufficient candidate instruments) (8). In the case of the reanalyzed Immunochip dataset, the same nominal threshold *p*-value was used (**Supplementary Table 1**) given the reduction in the sample size from 24,000 individuals to less than 5,000. Independent IVs were selected using the strict default clumping parameters (r^2^<0.001, window>10 000kb) of the *clump_data()* function. An *in-house* script was developed to extract the IVs from the GM and the HR-HLA CeD datasets to be used as outcome data (**Supplementary Data 1**).

Both exposure and outcome data were harmonized using the *harmonise_data()* function to guarantee that the effect that a given SNP has over the exposure and the outcome correspond to the same allele. After harmonization, the 2SMR analysis was completed using the different methods (Inverse Variance Weighted (IVW), MR Egger (MRE), Weighted Median (WMed), Weighted Mode (WMod), and Simple Mode (SMod)) available for the *mr()* function. Scatter plots were generated using the *mr_scatter_plot()* function, and forest plots with the *mr_forest_plot()* function.

## 3 Results

### 3.1 CeD susceptibility variants identified in individuals with high-risk HLA

In this study, we aimed to identify the reciprocal causality between the GM and CeD in HR-HLA individuals using a bidirectional 2SMR approach. Before performing the bidirectional 2SMR analysis, the Immunochip CeD genomic study (29) was reanalyzed considering only HR-HLA individuals (4) (**Supplementary Table S1**; **Supplementary Figure S1**). As shown in the Manhattan plot in **Supplementary Figure S1**, we obtained three association signals at genome-wide significance (*p*-value<5×10^−8^): the top signal maps to the *MHC* region on chromosome 6 (rs3128927, *p*-value=1.35×10^−14^), the second signal is on gene *PUS10* on chromosome 2 (rs10191951, *p*-value=8.41×10^−10^), and a novel association signal was located near *ATP23* gene on chromosome 12 (rs73344397, *p*-value=5.77×10^−9^). Along with these three regions, two additional signals corresponding to unique SNPs in those *loci* can be observed on chromosome 8 (rs138705229, *p*-value=4.25×10^-8^), and on chromosome 10 (rs375414915, *p*-value=2.53×10^-8^).

### 3.2 Bidirectional 2SMR identifies bacterial taxonomies causally related to high-risk HLA CeD

In order to explore whether the GM composition contributes to the development of CeD in HR-HLA individuals, or if on the contrary the disease modifies the composition of the GM in those individuals, we performed a 2SMR analysis in both directions: GM to HR-HLA CeD and HR-HLA CeD to GM (**Table 1**). As a first approach, the IVW method was used as an estimator of the potential causal effect that the exposure had on the outcome. In the GM to HR-HLA CeD direction (**Table 1**, upper panel), four microbial taxa from the phylum Firmicutes were identified, whereas in the HR-HLA CeD to GM direction (**Table 1**, lower panel), six microbial taxa from phyla Firmicutes, Bacteroidetes, and Proteobacteria resulted significant (IVW *p*-value<0.05).

**Table 1:** Summary of the potential causal associations between GM and HR HLA CeD.

GM to HR-HLA CeD								
TAXONOMIC LEVEL					2SMR RESULTS			
Phylum	Class	Order	Family	Genus	IVs	Beta	SE	*p*-value
Firmicutes	Clostridia	Clostridiales	*Ruminococcaceae*	*Ruminococcaceae UCG010*	3	1,690	0,828	0,041
Firmicutes	Clostridia	Clostridiales	*Ruminococcaceae*	*Ruminococcaceae UCG011*	2	-1,190	0,509	0,019
Firmicutes	Clostridia	Clostridiales	*Lachnospiraceae*	*Lachnospiraceae UCG008*	3	1,462	0,586	0,012
Firmicutes	Negativicutes	Veillonellales	*Veillonellaceae*	*-*	7	1,112	0,454	0,014
–	–	–	–	*FamilyXIII UCG001*	4	1,216	0,580	0,036
HR-HLA CeD to GM								
TAXONOMIC LEVEL					2SMR RESULTS			
Phylum	Class	Order	Family	Genus	IVs	Beta	SE	*p*-value
Firmicutes	Clostridia	Clostridiales	*Ruminococcaceae*	*Anaerotruncus*	3	-0,053	0,018	0,003
Firmicutes	Clostridia	Clostridiales	*Lachnospiraceae*	*Tyzzerella3*	3	-0,084	0,032	0,008
Bacteroidetes	Bacteroidia	Bacteroidales	*Rikenellaceae*	*RikenellaceaeRC9 gutgroup*	3	0,085	0,040	0,036
Proteobacteria	Gammaproteobacteria	–	–	*-*	3	0,047	0,018	0,011
Proteobacteria	Gammaproteobacteria	Pasteurellales	–	*-*	3	0,049	0,024	0,041
Proteobacteria	Gammaproteobacteria	Pasteurellales	*Pasteurellaceae*	*-*	3	0,049	0,024	0,041
–	–	–	–	*Unknowngenus*	3	-0,057	0,023	0,014

Significant threshold was set at *p*-value <0.05 for the Inverse Variance Weighted method (IVW). IVs, Instrumental Variables; SE, standard error.

In black are depicted the direct results from our bidirectional 2SMR analysis, while in grey are indicated the upper taxonomic level of each hit.

We next analyzed which of the bacterial taxa that resulted significant were common to both or specific to one direction. Some bacterial taxonomies observed, specifically the *Ruminococcaceae* and *Lachnospiraceae* families from the phylum Firmicutes (class Clostridia, order Clostridiales), are common to both directions, suggesting that they might play a role both as a cause and as a consequence of the disease. Other taxa only resulted significant in one of the two directions analyzed. In the GM to HR-HLA CeD direction, the class Negativicutes (*p*-value=0.014, beta=1.112 ± 0.454) resulted significant, suggesting a causal role of this taxon in the pathogenesis of HR-HLA CeD. Regarding the HR-HLA CeD to GM direction, classes Bacteroidia (*p*-value=0.036, beta=0.085 ± 0.040) or Gammaproteobacteria (*p*-value=0.041, beta 0.049 ± 0.024) gave a significant result, suggesting that their alteration could be a consequence rather than a cause of the disease itself. Of note, three of the seven hits in this direction are phylogenetically related to each other (class Gammaproteobacteria, order Pasteurellales, family *Pasteurellaceae*), highlighting their relevance in this direction. *FamilyXIII UCG001* and *unknowngenus* were excluded from subsequent analyses as there is no further information available.

### 3.3 *Veillonellaceae* and *Pasteurellaceae* families might play specific causal roles in the interplay between the GM and high-risk HLA CeD

**Figure 2:**
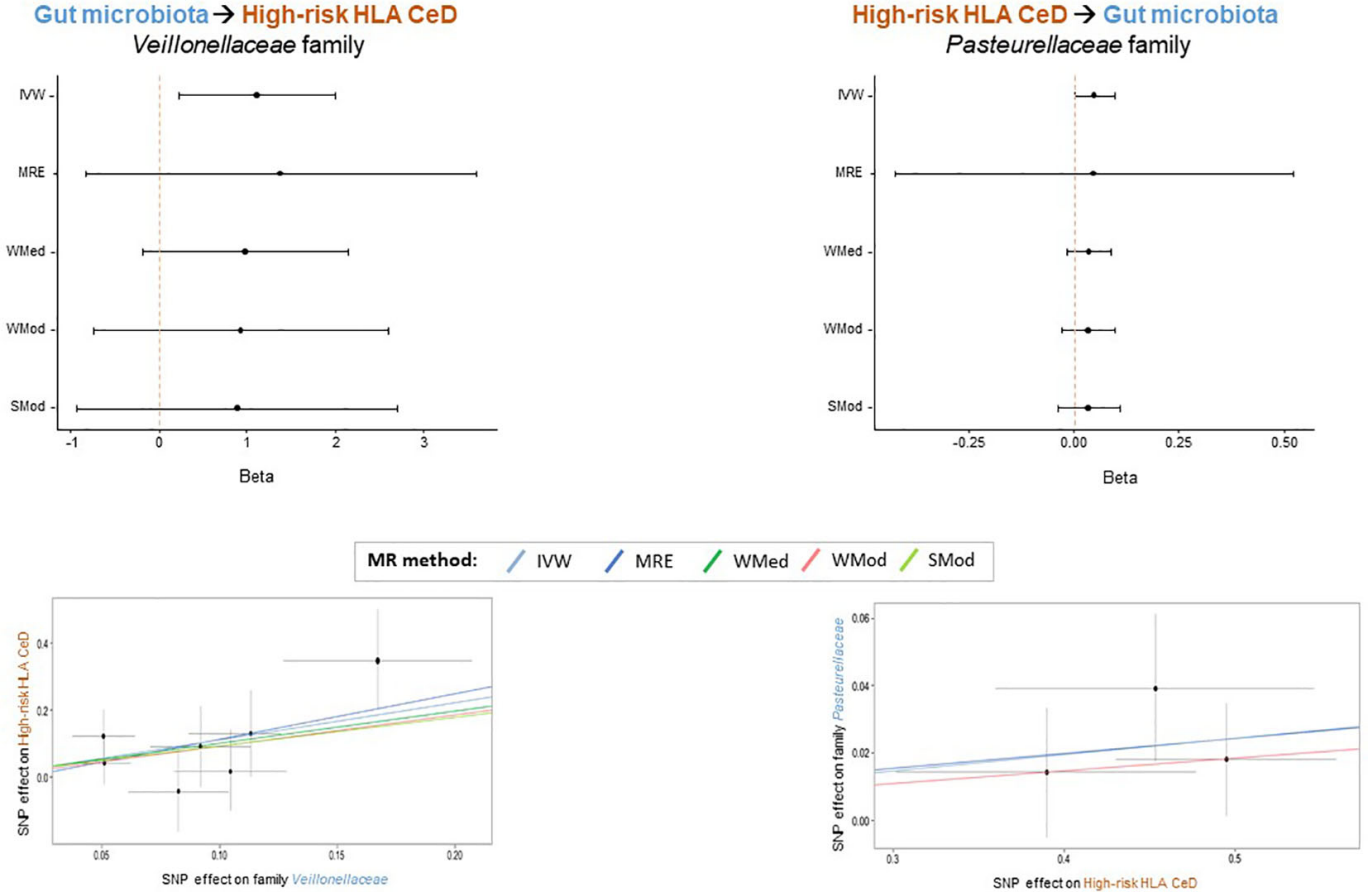
Bacterial taxa that might play a specific causal role in the interplay between GM and HR HLA CeD. Forest plots (upper panels) depict the consistency of the effect obtained across the 2SMR methods. Scatter plots (lower panels) show the consistent positive beta effect obtained with each method: Inverse Variance Weighted (IVW), MR Egger (MRE), Weighted Median (WMed), Weighted Mode (WMod), and Simple Mode (SMod).

In order to prioritize the most relevant hits, in addition to the IVW method, four additional methods from the 2SMR package were applied (MRE, WMed, WMod and SMod). Even if the *p-*values were not significant for all methods (**Supplementary Table S2**), forest plots illustrate a consistent direction in the effect for families *Veillonellaceae* (IVW beta=1.112; MRE beta=1.362; WMed beta=0.988; WMod beta=0.930; SMod beta=0.891) and *Pasteurellaceae* (IVW beta=0.049; MRE beta=0.045; WMed beta=0.037; WMod beta=0.037; SMod beta=0.037) in the GM to HR-HLA CeD and HR-HLA CeD to GM directions, respectively (**Figure 2**, upper panels). The absolute beta values were also very similar within each family, as evidenced in the slopes of the lines depicted in the scatter plots (**Figure 2**, lower panels). The consistency in the effect trends supports the causal role that each bacterial taxon might have in each direction, as will be discussed in the next section.

## 4 Discussion

An association between GM and CeD has been suggested in observational studies (11–13, 18–22). In this work, we evaluate for the first time the potential causality and directionality of this association in the context of the HR-HLA haplotypes by employing a bidirectional 2SMR approach. A previous study in our group also evaluated the interplay between host genetics, GM and CeD using a single-SNP 2SMR approach (25). This new analysis differs from the previous one in several aspects. Our current analysis follows a global MR approach that, using several SNPs as genetic instruments, allows the identification of causal associations between gut microbial taxa and HR-HLA CeD. In addition, this time we not only assess the potential causality that microbial alterations might have over the development of CeD, but also look at the causal consequences that a celiac gut might exert over the GM. Other more recent reports also employ a bidirectional 2SMR approach to evaluate the reciprocal causality between gut microbial genera and several diseases, including type 1 diabetes and CeD (8, 26–28). However, the current study is specifically focused on individuals at high genetic risk of developing CeD.

Our analysis identified bacteria from *Ruminococcaceae* and *Lachnospiraceae* families of phylum Firmicutes as potentially causal in both directions. Firmicutes is one of the most frequently altered phyla not only in CeD patients (15), but also in those individuals at high genetic risk of developing the disease (21, 22). Specifically, family *Ruminococcaceae* was identified in a recent genomic study as potentially causal in the GM to CeD direction (26). Our results are in line with previous literature and go further by suggesting that GM alteration can also be a consequence of the disease. In line with this observation, a study characterizing the gut microbiota of children with CeD found alterations in Ruminococcus fiber-fermenters between treated and untreated individuals, suggesting that their modulation is a consequence of the treatment with a GFD (17). With regard to the *Lachnospiraceae*, a family that belongs to the core GM, the impact in host physiology is controversial. Some studies have identified associations between different taxa of *Lachnospiraceae* family with several intra- and extra intestinal diseases (36), including CeD. In this context, a recent study has reported that CeD patients in GFD had a decreased level of three taxa belonging to *Lachnospiraceae* family (37). A randomized controlled trial resulted in both decreases and increases in the abundances of different species of the *Lachnospiraceae* family during a low-gluten diet intervention (38). Thus, even if family *Lachnospiraceae* appears as potentially causal in both directions, it is more likely that the directionality is determined at a lower taxonomic level such as genus or species. Another possible interpretation of the potential dual role that *Ruminococcaceae* and *Lachnospiraceae* families have might come from a context-specific interaction, where depending on environmental factors, these bacteria might trigger the development of the disease, or be the consequence of a celiac gut microenvironment.

According to our data, other bacterial taxa might play a specific causal role in each of the directions analyzed. This is the case for the *Veillonellaceae* and *Pasteurellaceae* families, which yielded significant results with the IVW method in the GM to HR-HLA CeD and HR-HLA CeD to GM directions, respectively. Moreover, these significant findings were consistent across sensitivity analyses, and all the methods showed concordant effects, further supporting their specific causal role in each direction. On the one hand, our 2SMR analysis suggested that an increased abundance of family *Veillonellaceae* might have a causal effect on HR-HLA CeD. This potential causality might be extended to other gut diseases such as Crohn’s disease, where an increased abundance of these type of pro-inflammatory bacteria has been consistently observed (39, 40). In addition, a decrease in *Veillonellaceae* abundance has been reported in healthy individuals following a GFD (16). In addition to the protective effect that gluten deprivation has over CeD, our results could suggest that a GFD might slightly contribute through the reduction of *Veillonellaceae* bacteria. However, we must take into account that the reduction in *Veillonellaceae* abundance was observed in healthy volunteers, and not in individuals at high genetic risk of developing CeD. On the other hand, a higher genetic risk for CeD was predicted to increase the abundance in several taxa belonging to phylum Proteobacteria, specifically class Gammaproteobacteria, order Pasteurellales and family *Pasteurellaceae*. Interestingly, the phylum Proteobacteria has been reported to be more abundant not only in active celiac individuals compared to GFD-treated celiac patients and controls (41), but also in CeD individuals experiencing persistent symptoms, despite showing a normal histology and adhering to a GFD (15). It is conceivable to think that the higher abundance of Proteobacteria observed in these individuals could be a consequence rather than a cause of the disease itself.

Nevertheless, some limitations of our study should be considered. The first one is related to the small number of HLA-DQ2 non-celiac controls (n=550) compared to the celiac group (n=4,264) in the Immunochip study (29). However, this is an intrinsic limitation of the study design, as we intentionally selected those people with the highest genetic risk of developing CeD, which considerably increases the proportion of celiac individuals and restrains that of healthy controls. The second limitation relates to the ancestry of the participants in the Immunochip study. In our analysis, we used data of individuals mainly from European ancestry, where the HLA-DQ2 is the predominant risk haplotype. However, studies in Latin America indicate that HLA-DQ8 could confer a higher risk to CeD, and this might also modify the microbiota composition. It would be interesting to carry out a similar study using GWAS datasets from other ancestries to ascertain whether our findings can be generalized to other ethnic groups. The third limitation is related to the high inter-individual microbial variability observed across the 24 cohorts from the MiBioGen study (8), which reduces the statistical power of these kind of multi-cohort meta-analyses. Very recently, Qin et al. published a new microbiome GWAS in a single cohort of almost 6,000 European individuals, identifying genetic variants associated with certain microbes (29). The top genetic *loci* identified to be associated with bacterial taxa remain the same as in the MiBioGen study, suggesting that the strategy of using a single homogeneous cohort lies behind the constraint on the sample size. Fourth, the taxonomic resolution of the MiBioGen database reaches the genus level, leaving out more accurate genetic associations at the species or strain levels, and reports some uncharacterized hits. Thus, it would be interesting to repeat this 2SMR analysis when more specialized microbiota GWAS become available. To finalize, although in this study we have investigated the bidirectional causality between GM and CeD in high-risk individuals, microbiota from other niches such as the breast milk have been suggested to protect from autoimmune diseases, including CeD (6). Thus, it could be interesting to investigate the potential protective role of breastfeeding in a genetic setting predisposing to CeD. In summary, this is the first study that evaluates the bidirectional causality between GM composition and CeD in the context of the HR-HLA haplotype using a 2SMR approach. We not only identify a set of microbial taxa that might be involved in the pathogenesis of CeD, but also identify other bacteria whose altered abundance might be a consequence of the disease itself. Our results, although preliminary and pending validation, could give clues to highlight potential focus on future research in the field of GM and CeD. Moreover, our computational approach could be applied to study other autoimmune diseases such as type 1 diabetes.

## Data availability statement

The original contributions presented in the study are included in the article/**Supplementary Materials**, further inquiries can be directed to the corresponding author/s.

## Ethics statement

The data was based on published studies or databases and no additional ethical approval from an institutional review board was required.

## Author contributions

JR-B designed the research. BG-G and SM collected the data. SM wrote the initial scripts, and BG-G performed the global analyses. AC-P and AH-L helped in the analyses. BG-G and IG-S performed the literature search. BG-G and IG-S drafted the article. NF-J revised the first draft and gave feedback. IG-S and JR-B supervised the study. All authors were involved in writing the paper. All authors contributed to the article and approved the submitted version.

## Supplementary material

Supplementary Figure 1 Manhattan plot of the Immunochip Association Study (GWAS) of HR-HLA individuals. Each dot represents a variant plotted as −*log* 10 (*p*-value) on the *y*-axis against the corresponding variant position on the *x*-axis. The red line represents the genome-wide significance threshold value (*p*-value<5x10^-8^), and the blue line represents the nominal significance threshold (*p*-value<1x10^-5^).

Supplementary Table 1 Independent genetic variants below 1x10^-5^ nominal significance level in the HR-HLA CeD association analysis.

Supplementary Table 2 Results of the bidirectional 2SMR analysis with the five methods in both directions. HR-HLA CeD: High-risk HLA Celiac Disease; GM: Gut Microbiota; IVs: Instrumental Variables; SE: standard error; *p-*val: *p*-value.
