# Supplementary figures and images for: Two-Sample Mendelian Randomization detects bidirectional causality between gut microbiota and celiac disease in individuals with high genetic risk
Source: Front Immunol. 2023 Jun 30;14:1082862. doi: 10.3389/fimmu.2023.1082862 (PMC10347381; doi:10.3389/fimmu.2023.1082862)

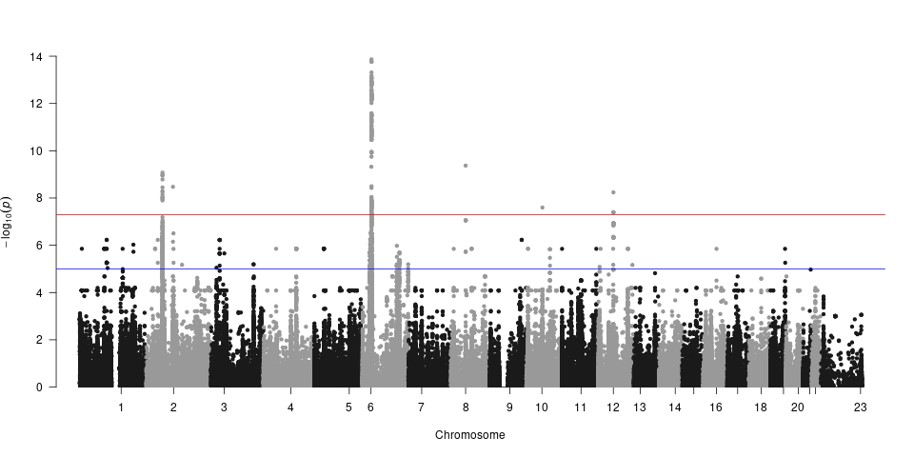

Supplement: Supplementary Figure 1 — Manhattan plot of the Immunochip Association Study (GWAS) of HR-HLA individuals. Each dot represents a variant plotted as −log 10 (p-value) on the y-axis against the corresponding variant position on the x-axis. The red line represents the genome-wide significance threshold value (p-value<5x10^-8^), and the blue line represents the nominal significance threshold (p-value<1x10^-5^). [file Image_1.jpeg]
