# Supplementary material for: Two-Sample Mendelian Randomization detects bidirectional causality between gut microbiota and celiac disease in individuals with high genetic risk
Source: Front Immunol. 2023 Jun 30;14:1082862. doi: 10.3389/fimmu.2023.1082862 (PMC10347381; doi:10.3389/fimmu.2023.1082862)
Supplement: Supplementary Table 2 — Results of the bidirectional 2SMR analysis with the five methods in both directions. HR-HLA CeD: High-risk HLA Celiac Disease; GM: Gut Microbiota; IVs: Instrumental Variables; SE: standard error; p-val: p-value. [file DataSheet_2.docx]

| **GM to HR-HLA CeD** | | | | | | | | | | | | | | | | |
| --- | --- | --- | --- | --- | --- | --- | --- | --- | --- | --- | --- | --- | --- | --- | --- | --- |
|  |  | **Inverse variance weighted** | | | **Weighted median** | | | **MR Egger** | | | **Simple mode** | | | **Weighted mode** | | |
| **Taxonomic unit** | **IVs** | **Beta** | **SE** | ***p-*val** | **Beta** | **SE** | ***p-*val** | **Beta** | **SE** | ***p-*val** | **Beta** | **SE** | ***p-*val** | **Beta** | **SE** | ***p-*val** |
| g_Lachnospiraceae UCG008 | 3 | 1.462 | 0.586 | 0.013 | 1.319 | 0.762 | 0.083 | -1.797 | 4.555 | 0.761 | 1.342 | 0.922 | 0.283 | 1.263 | 0.856 | 0.278 |
| f_Veillonellaceae | 7 | 1.112 | 0.454 | 0.014 | 0.988 | 0.619 | 0.110 | 1.362 | 1.127 | 0.281 | 0.891 | 0.938 | 0.379 | 0.930 | 0.986 | 0.382 |
| g_Ruminococcaceae UCG011 | 2 | -1.190 | 0.509 | 0.019 | Not enough IVs | | | Not enough IVs | | | Not enough IVs | | | Not enough IVs | | |
| g_FamilyXIII UCG001 | 4 | 1.216 | 0.580 | 0.036 | 1.376 | 0.718 | 0.055 | 1.443 | 2.341 | 0.600 | 1.492 | 1.091 | 0.265 | 1.503 | 1.029 | 0.240 |
| g_Ruminococcaceae UCG010 | 3 | 1.690 | 0.828 | 0.041 | 1.078 | 1.043 | 0.301 | -2.045 | 4.266 | 0.715 | 0.803 | 1.156 | 0.559 | 0.775 | 1.264 | 0.602 |
|  |  |  |  |  |  |  |  |  |  |  |  |  |  |  |  |  |
| **HR-HLA CeD to GM** | | | | | | | | | | | | | | | | |
|  |  | **Inverse variance weighted** | | | **Weighted median** | | | **MR Egger** | | | **Simple mode** | | | **Weighted mode** | | |
| **Taxonomic unit** | **IVs** | **Beta** | **SE** | ***p-*val** | **Beta** | **SE** | ***p-*val** | **Beta** | **SE** | ***p-*val** | **Beta** | **SE** | ***p-*val** | **Beta** | **SE** | ***p-*val** |
| g_Anaerotruncus | 3 | -0.053 | 0.018 | 0.003 | -0.051 | 0.023 | 0.022 | 0.050 | 0.183 | 0.829 | -0.051 | 0.028 | 0.212 | -0.051 | 0.025 | 0.173 |
| g_Tyzzerella3 | 3 | -0.084 | 0.032 | 0.008 | -0.087 | 0.037 | 0.018 | 0.101 | 0.319 | 0.805 | -0.109 | 0.048 | 0.152 | -0.060 | 0.040 | 0.276 |
| c_Gammaproteobacteria | 3 | 0.047 | 0.018 | 0.011 | 0.043 | 0.022 | 0.049 | 0.182 | 0.185 | 0.506 | 0.031 | 0.028 | 0.385 | 0.062 | 0.024 | 0.126 |
| g_unknowngenus | 3 | -0.057 | 0.023 | 0.015 | -0.055 | 0.029 | 0.059 | -0.345 | 0.233 | 0.379 | -0.035 | 0.039 | 0.462 | -0.073 | 0.031 | 0.146 |
| g_Rickenellacear RC9gut group | 3 | 0.085 | 0.040 | 0.036 | 0.068 | 0.049 | 0.164 | -0.308 | 0.408 | 0.588 | 0.054 | 0.057 | 0.448 | 0.060 | 0.055 | 0.383 |
| f_Pasteurellaceae | 3 | 0.049 | 0.024 | 0.041 | 0.037 | 0.030 | 0.213 | 0.045 | 0.241 | 0.883 | 0.037 | 0.036 | 0.411 | 0.037 | 0.032 | 0.365 |
| o_Pasteurellales | 3 | 0.049 | 0.024 | 0.041 | 0.037 | 0.027 | 0.169 | 0.045 | 0.241 | 0.883 | 0.037 | 0.033 | 0.396 | 0.037 | 0.031 | 0.361 |
