# Supplementary material for: Two-Sample Mendelian Randomization detects bidirectional causality between gut microbiota and celiac disease in individuals with high genetic risk
Source: Front Immunol. 2023 Jun 30;14:1082862. doi: 10.3389/fimmu.2023.1082862 (PMC10347381; doi:10.3389/fimmu.2023.1082862)
Supplement: Supplementary file 4 [file DataSheet_3.docx]

#Two Sample Mendelian Randomization detects bidirectional causality between gut

#microbiota and celiac disease in individuals with high genetic risk

#BP.Gonzalez-Garcia

#October 2022

#UPV/EHU

#Please update files and paths accordingly

#===============================================================================#

# HR-HLA CeD to GM

# Exposure data: HR-HLA CeD Outcome data: GM

#===============================================================================#

#Install packages

install.packages('devtools')

install_github("MRCIEU/TwoSampleMR")

install.packages('R.utils')

library(devtools)

library(data.table)

library(TwoSampleMR)

library(ggplot2)

library(data.table)

#Set working directory

setwd("~/MICROBIOME/cedmicro")

#-------------------------------------------------------------------------------

#1)Load SNP test output and match with 2SMR requirements

gwas_dq2 <- read.table('immuno_dq2.out', header = T)

#Calculate effect allele frequency

gwas_dq2$eaf <- (gwas_dq2$all_BB + (gwas_dq2$all_AB/2)) / gwas_dq2$all_total

#Change column names accordingly to 2SMR requirements

colnames(gwas_dq2)

names(gwas_dq2)[2:6] <- c("SNP", "chr", "position", "other_allele", "effect_allele")

names(gwas_dq2)[18] <- c("samplesize")

names(gwas_dq2)[23] <- c("ncase")

names(gwas_dq2)[28] <- c("ncontrol")

names(gwas_dq2)[42] <- c("pval")

names(gwas_dq2)[44:45] <- c("beta", "se")

names(gwas_dq2)[47] <- c("eaf")

#Remove NA in pvalues

gwas_dq2 <- gwas_dq2[!is.na(gwas_dq2$pval), ]

#-------------------------------------------------------------------------------

#2)Prepare exposure data - HR-HLA CeD

exp_formatted_dq2 <- format_data(gwas_dq2,

type = 'exposure',

#phenotype_col = 'Phenotype',

snp_col = 'SNP',

beta_col = 'beta',

se_col = 'se',

eaf_col = 'eaf',

effect_allele_col = 'effect_allele',

other_allele_col = 'other_allele',

pval_col = 'pval',

samplesize_col = 'samplesize',

chr_col = 'chr',

pos_col = 'position')

#Filter by p-value 1e-5 and default clumping (r2=0.001)

exp_sig_dq2 <- exp_formatted_dq2[exp_formatted_dq2$pval.exposure < 1e-5, ]

exp_sig_dq2 <- clump_data(exp_sig_dq2)

#-------------------------------------------------------------------------------

#3)Prepare outcome data - GM

makeOutcome <- function(filename, exposure) {

cat('Formating', crayon::magenta(filename), '... ')

outcome <- fread(filename)

#Using file name substring as phenotype

pheno <- tail(strsplit(filename, '/')[[1]], 1)

pheno <- gsub('.summary.txt.gz', '', pheno)

pheno <- gsub('.txt', '', pheno)

outcome[, phenotype := pheno]

#Outcome data format

outcome <- format_data(outcome,

type = 'outcome',

phenotype_col = 'phenotype',

snps = exposure$SNP,

snp_col = 'rsID',

beta_col = 'beta',

se_col = 'SE',

effect_allele_col = 'eff.allele',

other_allele_col = 'ref.allele',

pval_col = 'P.weightedSumZ',

samplesize_col = 'N',

z_col = 'Z.weightedSumZ',

chr_col = 'chr',

pos_col = 'bp')

cat(crayon::green('Done!'), '\n')

return(outcome)

}

#List of all paths

phylum <- list.files('/data/Genomics_projects/micro2smr/data/mibiogen/phylum', '.txt.gz', full.names = T)

class <- list.files('/data/Genomics_projects/micro2smr/data/mibiogen/class', '.txt.gz', full.names = T)

order <- list.files('/data/Genomics_projects/micro2smr/data/mibiogen/order', '.txt.gz', full.names = T)

family <- list.files('/data/Genomics_projects/micro2smr/data/mibiogen/family', '.txt.gz', full.names = T)

genus <- list.files('/data/Genomics_projects/micro2smr/data/mibiogen/genus', '.txt.gz', full.names = T)

all_files <- c(phylum, class, order, family, genus)

outcome_dq2 <- lapply(all_files, makeOutcome, exposure = exp_sig_dq2)

outcome_dq2 <- rbindlist(outcome_dq2)

#-------------------------------------------------------------------------------

#4)Harmonise data

harmonised_dq2 <- harmonise_data(exposure_dat = exp_sig_dq2, outcome_dat = outcome_dq2)

#-------------------------------------------------------------------------------

#5)Run 2SMR

res_dq2 <- mr(harmonised_dq2)

#-------------------------------------------------------------------------------

#6)Scatter plots

#Create scatter plot with lines showing the causal estimate for different MR tests

#mr_scatter_plot(mr_results, dat)

#-------------------------------------------------------------------------------

#7)Forest plot

#Calculate upper and lower levels

resPF8PPj$ul <- (resPF8PPj$b+(1.96*resPF8PPj$se))

resPF8PPj$ll <- (resPF8PPj$b-(1.96*resPF8PPj$se))

resPF8PPj$index <- 1:nrow(resPF8PPj)

#Graph

ggplot(data = resPF8PPj,aes(y=index, x=b, xmin=ll, xmax=ul)) +

geom_point() +

geom_errorbarh(height = 0.1) +

scale_y_continuous(breaks = 1:nrow(resPF8PPj), labels = resPF8PPj$method) +

labs(title= 'f_Pasteurellaceae', x='Effect size', y= 'Methods') +

geom_vline(xintercept = 0.0, color='red', linetype= 'dashed', alpha=0.5) +

theme_classic()

#===============================================================================#

# GM to HR-HLA

# Exposure data: GM Outcome data: HR-HLA CeD

#===============================================================================#

#1)Prepare exposure data - GM

exposureMiBioGen <- fread('/data/Genomics_projects/micro2smr/data/mibiogen/MBG.allHits.p1e4.txt')

#Exposure data format

expMiBioGen_formatted <- format_data(exposureMiBioGen,

type = 'exposure',

phenotype_col = 'bac',

snp_col = 'rsID',

beta_col = 'beta',

se_col = 'SE',

effect_allele_col = 'eff.allele',

other_allele_col = 'ref.allele',

pval_col = 'P.weightedSumZ',

samplesize_col = 'N',

z_col = 'Z.weightedSumZ',

chr_col = 'chr',

pos_col = 'bp')

#-------------------------------------------------------------------------------

#2)Perform clumping

expMiBioGen_clumped <- clump_data(expMiBioGen_formatted)

#-------------------------------------------------------------------------------

#3)Prepare outcome data: HR-HLA

makeOutcomeHLA <- function(filename, exposure) {

cat('Formating', crayon::magenta(filename), '... ')

# Outcome data format

outcome <- fread(filename,

select = c('rsid', 'chromosome', 'position', 'alleleA', 'alleleB',

'all_AA', 'all_AB', 'all_BB', 'all_total',

'frequentist_add_pvalue', 'frequentist_add_beta_1',

'frequentist_add_se_1', 'cases_total', 'controls_total'),

showProgress = F

)

# pheno <- tail(strsplit(filename, '/')[[1]], 1)

# pheno <- gsub('_allchr.txt.gz', '', pheno)

# outcome[, phenotype := pheno]

outcome[, eaf := (all_BB + (all_AB/2)) / all_total]

outcome <- format_data(outcome,

type = 'outcome',

# phenotype_col = 'phenotype',

snps = exposure$SNP,

snp_col = 'rsid',

beta_col = 'frequentist_add_beta_1',

se_col = 'frequentist_add_se_1',

eaf_col = 'eaf',

effect_allele_col = 'alleleB',

other_allele_col = 'alleleA',

pval_col = 'frequentist_add_pvalue',

samplesize_col = 'all_total',

chr_col = 'chromosome',

pos_col = 'position',

ncase= 'cases_total',

ncontrol= 'controls_total')

cat(crayon::green('Done!'), '\n')

return(outcome)

}

outcome_dq2MBG <- makeOutcomeHLA('immuno_dq2.out', expMiBioGen_clumped)

#-------------------------------------------------------------------------------

#4)Harmonise data

harmonised_dq2MBG <- harmonise_data(exposure_dat = expMiBioGen_clumped, outcome_dat = outcome_dq2MBG)

#-------------------------------------------------------------------------------

#5)Run 2SMR

res_dq2MBG <- mr(harmonised_dq2MBG)

#-------------------------------------------------------------------------------

#6)Scatter plots

scatter_plotMBG<-mr_scatter_plot(res_dq2MBG,harmonised_dq2MBG)

#-------------------------------------------------------------------------------

#7) Forest plot

#Calculate upper and lower levels

resYBKuOp$ul <- (resYBKuOp$b+(1.96*resYBKuOp$se))

resYBKuOp$ll <- (resYBKuOp$b-(1.96*resYBKuOp$se))

resYBKuOp$index <- 1:nrow(resYBKuOp)

#Graph

ggplot(data = resYBKuOp,aes(y=index, x=b, xmin=ll, xmax=ul)) +

geom_point() +

geom_errorbarh(height = 0.1) +

scale_y_continuous(breaks = 1:nrow(resYBKuOp), labels = resYBKuOp$method) +

labs(title= 'Microbiome -> Celiac disease "Veillonellaceae"', x='beta', y= 'Method') +

geom_vline(xintercept = 0.0, color='red', linetype= 'dashed', alpha=0.5) +

theme_classic()
